# Risk factors, costs and complications of delayed hospital discharge from internal medicine wards at a Canadian academic medical centre: retrospective cohort study

**DOI:** 10.1186/s12913-019-4760-3

**Published:** 2019-12-04

**Authors:** Anthony D. Bai, Cathy Dai, Siddhartha Srivastava, Christopher A. Smith, Sudeep S. Gill

**Affiliations:** 1 Department of Medicine, Queen’s University, Kingston, Ontario Canada; 2 Kingston Health Sciences Centre, Kingston, Ontario Canada; 3 Providence Care Hospital, Kingston, 752 King St. West, Kingston, ON K7L 4X3 Canada

**Keywords:** General internal medicine, Delayed discharge, Clinical prediction rule

## Abstract

**Background:**

Hospitalized patients are designated alternate level of care (ALC) when they no longer require hospitalization but discharge is delayed while they await alternate disposition or living arrangements. We assessed hospital costs and complications for general internal medicine (GIM) inpatients who had delayed discharge. In addition, we developed a clinical prediction rule to identify patients at risk for delayed discharge.

**Methods:**

We conducted a retrospective cohort study of consecutive GIM patients admitted between 1 January 2015 and 1 January 2016 at a large tertiary care hospital in Canada. We compared hospital costs and complications between ALC and non-ALC patients. We derived a clinical prediction rule for ALC designation using a logistic regression model and validated its diagnostic properties.

**Results:**

Of 4311 GIM admissions, 255 (6%) patients were designated ALC. Compared to non-ALC patients, ALC patients had longer median length of stay (30.85 vs. 3.95 days *p* < 0.0001), higher median hospital costs ($22,459 vs. $5003 *p* < 0.0001) and more complications in hospital (25.5% vs. 5.3% *p* < 0.0001) especially nosocomial infections (14.1% vs. 1.9% *p* < 0.0001). Sensitivity analyses using propensity score and pair matching yielded similar results. In a derivation cohort, seven significant risk factors for ALC were identified including age > =80 years, female sex, dementia, diabetes with complications as well as referrals to physiotherapy, occupational therapy and speech language pathology. A clinical prediction rule that assigned each of these predictors 1 point had likelihood ratios for ALC designation of 0.07, 0.25, 0.66, 1.48, 6.07, 17.13 and 21.85 for patients with 0, 1, 2, 3, 4, 5, and 6 points respectively in the validation cohort.

**Conclusions:**

Delayed discharge is associated with higher hospital costs and complication rates especially nosocomial infections. A clinical prediction rule can identify patients at risk for delayed discharge.

## Background

A growing proportion of patients experience delayed hospital discharge after they are deemed medically appropriate to be discharged from hospital [1]. Delayed discharge is often driven by functional dependence requiring increased assistance or alternative living arrangements (e.g. nursing home placement), patient or family disagreement with discharge plans, availability of community resources, and inadequate social supports [2]. Delayed discharge is a prevalent issue across hospitals in many countries including Canada [3], the United States [2, 4], and England [5]. In Canada, hospitalized patients with delayed discharge are designated alternate level of care (ALC) [6].

ALC status is assigned to a patient who continues to occupy a hospital bed when they no longer require the intensity of resources and services provided in that care setting. This is decided based on the attending physician’s judgment. Other than not being rounded on a daily basis by the physician, the care of ALC patients are mostly identical to non-ALC patients in hospital. ALC status is mainly used by hospital systems to identify patients with delayed discharges. With the exception of patients designated as awaiting long-term care, ALC designation does not directly impact physician billing. ALC patients who require chronic care and permanently reside in hospital or another institution may be asked for co-payment for meals and accommodation [7].

Delayed discharges have many negative impacts on patient safety, quality of care as well as health system utilization and costs. First, prolonged hospitalization increases risk for adverse outcomes including accelerated functional decline, delirium, pressure ulcers, nosocomial infections and falls [8–12]. Second, delayed discharge creates patient safety challenges by contributing to hospital overcrowding and reduced accessibility to finite acute care resources. This contributes to adverse outcomes from emergency department boarding and bedspacing [13]. Third, delayed discharge increases hospital and health system costs from inappropriate use of high cost hospital beds for care better provided in alternative settings (e.g. home care, long-term care) [3, 14]. Finally, unnecessary and prolonged hospital stay contributes to patient and family stress and stigmatization [15, 16].

To date, studies on delayed discharge have mainly focused on a large provincial level [17] or patients not on general internal medicine (GIM) wards [4, 18–21]. A few studies have used qualitative methods to examine the experience of ALC designation in small numbers of patients [15, 16]. No study to date has focused on GIM inpatients. GIM patients are a unique population and make up a significant proportion of all patients admitted to hospital, which was estimated to be approximately 40% [22].

Description of risk factors, cost and complications for delayed discharge is important. With this knowledge, hospitals can address modifiable risk factors. To the best of our knowledge, no study has been published that presents a clinical prediction rule for delayed discharge in the Canadian health system. From a hospital perspective, such a clinical prediction rule would be useful to identify high-risk patients as targets for preventative measures such as early anticipatory discharge planning. Description of the cost and complication rate better quantifies consumption of hospital resources and negative impact on quality of care.

The objectives of our retrospective cohort study were to describe the risk factors, cost and complications associated with delayed discharge for GIM patients. We also derived and validated a clinical prediction rule to identify GIM patients at high risk for delayed discharge.

## Methods

### Design

We conducted a retrospective cohort study using hospital data collected during the 2015 year at a large Canadian tertiary care hospital. The institution’s ethics committee granted approval for this study. The reporting is based on the RECORD guidelines for observational studies using routinely collected data [23].

### Setting

The study took place at a tertiary level, acute care, university affiliated teaching hospital with a catchment area exceeding 500,000 people. It has 440 inpatient beds and admits more than 22,000 patients annually.

### Patient population

We included consecutive adult patients admitted to the GIM service at the hospital from 1 January 2015 to 1 January 2016.

Patients were excluded if they were:

- Admitted from nursing homes
- Transferred from another acute care hospital for specialized care
- Transferred to another service during their hospital stay
- Left against medical advice
- Died within 48 h of hospital admission

Patients admitted to other acute care hospitals or from nursing homes were excluded, because they already have a designated discharge destination and not appropriate for ALC designation in most cases.

### Data sources

Data were obtained from patient electronic medical records and the Discharge Abstract Database, an administrative database collected by the Canadian Institute of Health Information (CIHI) for all hospital discharges [24].

### Alternate level of care (ALC) designation

In Ontario, ALC is uniformly defined as a patient who occupies a bed in a hospital and does not require the intensity of resources and services provided in that care setting [6]. The main responsible physician designates patients as ALC when the patients’ care goals have been met and they are awaiting discharge or transfer to destinations such as home with increased supports, rehabilitation hospitals, long term care home and retirement home.

### Variable definition

For patients, the estimated income was the median yearly income based on the forward sortation area (first 3 digits of the postal code) of patient homes and the 2016 Census by Statistics Canada [25].

Case mix groups (CMG) were based on CMG methodology as described by CIHI [26–29]. Admission diagnoses were organized into CMGs based on International Classification of Disease 10th Revision Canadian version (ICD-10-CA) codes [30]. CMG had a factor for each of the 52 most common groups, which accounted for approximately 80% of all admissions. ICD-10-CA diagnoses codes present at and after admission for each patient were used to calculate the Charlson Comorbidity Index (CCI) [31] using an established coding algorithm [32].

Referrals to physiotherapy, occupational therapy, speech language pathology and respiratory therapy anytime during hospitalization were recorded.

### Follow-up in hospital

Patients were followed until discharge from hospital. Variables collected at discharge included hospital costs, complications during hospital stay, in-hospital mortality and discharge destination.

Length of stay was number of days from admission to discharge. For ALC patients, ALC days were days that the patients were designated ALC during their hospital stay.

Estimated hospital cost per patient was based on the resource intensity weight (RIW), which is a weighted summary measure representing the relative value of resources a patient was expected to consume based on their age, CMG, comorbidities and length of stay [ 26–29]. Cost of a Standard Hospital Stay (CSHS) is the ratio of a hospital’s total acute inpatient care expenses to number of acute inpatient weighted cases [33]. A patient’s RIW multiplied by the hospital specific CSHS was equal to the estimated hospital cost of treating that patient [34].

Complications any time during hospitalization were recorded on the discharge summary by the treatment team. Complications were entered as events that occurred after hospital admission, which were distinct from comorbidities present prior to admission. We included common complications associated with extended hospital stay including delirium, aspiration, pulmonary embolism, acute decompensated heart failure, acute kidney injury, pressure ulcer, traumatic fractures, adverse drug effects and nosocomial infections. Nosocomial infections included pneumonia, urinary tract infection, *Clostridioides difficile* colitis and sepsis. Any complication refers to only the complications listed above.

All cause in-hospital mortality was recorded. Discharge destinations were categorized as other acute care hospital, rehabilitation centre, home, home with community agency support, retirement home, nursing home, chronic care and other. Readmission to the same hospital within 30 days of discharge was recorded.

### Statistical analyses

Descriptive analyses included median (interquartile range (IQR)) for continuous variables and number (percentage) for categorical variables. Continuous variables were compared using the Wilcoxon rank-sum test. Chi-square or Fisher exact test were used to compare categorical variables when appropriate.

As a sensitivity analysis, we conducted a matched pair analysis using propensity score matching. Details are provided in Additional file 1: Text S1.

The study population was randomly subdivided into equal-sized derivation and validation cohorts. In the derivation cohort, a logistic regression model was used to derive significant predictors for ALC designation. In the logistic regression model, ALC designation was the dependent variable. Potential predictors were chosen a priori and listed in study protocol during the study design stage by two authors (ADB and SSG). These predictors were entered as independent variables in the model. Potential predictors included age, sex, median yearly income, marital status, original placement, Charlson comorbidity categories, CCI score, CMG and need for allied health team. From univariate analyses, predictors were considered significant based on threshold *p*-value of < 0.2. Significant predictors were then entered into a multivariable logistic regression model. Forward and backward stepwise regression model based on Akike information criterion as well as clinical judgment were used to derive the final multivariable logistic regression. Clinical judgment was based on plausibility of the causal mechanism of how the predictor would contribute to delayed discharge. Four clinicians (ADB, SSG, CAS and SS) reviewed and reached consensus that the significant predictors in the final model were clinically relevant.

A clinical prediction rule was created based on the predictors in the final multivariable logistic regression model. For each predictor in the model, patients were given 1 point in the clinical prediction rule.

In the validation cohort, diagnostic properties were calculated using the clinical prediction rule as the test and ALC designation as the criterion standard. We calculated likelihood ratios and associated 95% confidence intervals (CI) based on method described by Simel et al. [35] A ROC curve was constructed based on the different cutoffs for the clinical prediction rule and an area under the curve (AUC) was calculated.

Our study data were complete without any missing data, so statistical handling of missing data was not applicable.

All reported CIs were two-sided 95% intervals and all tests were two-sided with a *P* < 0.05 significance level. All analyses were done with R V.3.4.3 (R Foundation for Statistical Computing, Vienna, Austria).

## Results

### Description of ALC and non-ALC patients

**Fig. 1:**
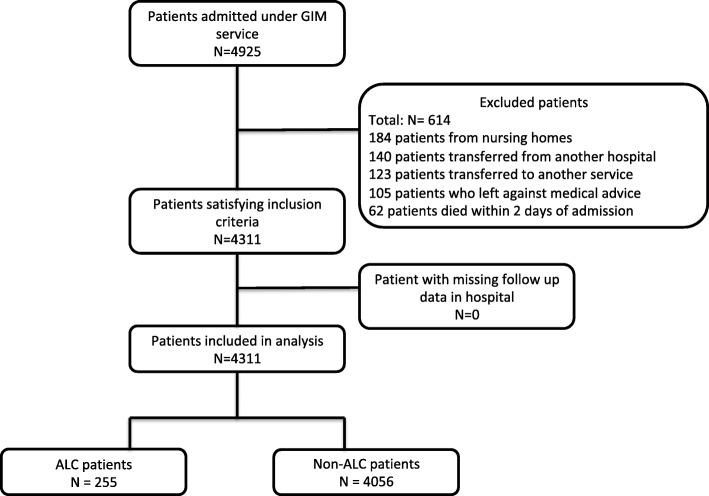
Flow diagram of included patients

Of 4311 patients, 255 (6%) patients were designed ALC during their hospital stay (Fig. 1, Table 1 and Additional file 1: Table S1). The median length of ALC designation was 13 days (IQR 7–37.5 days). The total number of ALC days was 9339 days, which was 24% of the total number of patient days for all admitted GIM inpatients. Time to physiotherapy, occupational therapy and speech language pathology are described in Additional file 1: Figure S1.

**Table 1:** Baseline characteristics and outcomes of ALC and non-ALC patients

	ALC patients (*N* = 255)	Non-ALC patients (*N* = 4056)	*P*-value
Age median (IQR)	81.0 (72.5–88.0)	68.0 (55.0–79.0)	< 0.0001
Age by category			
< 80 years	103 (40.4%)	3059 (75.4%)	< 0.0001
> =80 years	152 (59.6%)	997 (24.6%)	
Male	111 (43.5%)	2049 (50.5%)	0.0357
Estimated yearly income ($) based on postal code	35,277	35,277	0.4345
Median (IQR)	(32,410-36,685)	(31,883-36,685)	
Marital status			
Currently married	100 (39.2%)	1817 (44.8%)	< 0.0001
Widowed	87 (34.1%)	707 (17.4%)	
Other	68 (26.7%)	1532 (37.8%)	
Admitted from			
Home	187 (73.3%)	3639 (89.7%)	< 0.0001
Retirement home	51 (20.0%)	245 (6.0%)	
Other	17 (6.7%)	172 (4.2%)	
Charlson Comorbidity Index			
0	50 (19.6%)	1322 (32.6%)	< 0.0001
1	57 (22.4%)	941 (23.2%)	
> =2	148 (58.0%)	1793 (44.2%)	
Charlson Comorbidity			
Cerebrovascular disease	19 (7.5%)	83 (2.1%)	< 0.0001
Dementia	79 (31.0%)	184 (4.5%)	< 0.0001
Diabetes with complications	75 (29.4%)	790 (19.5%)	0.0002
Top 7 CMG			
139	11 (4.3%)	430 (10.6%)	0.0019
Chronic obstructive pulmonary disease			
138	15 (5.9%)	232 (5.7%)	> 0.9999
Viral / unspecified pneumonia			
487	17 (6.7%)	173 (4.3%)	0.0980
Lower urinary tract infection			
254	4 (1.6%)	145 (3.6%)	0.1090
Gastrointestinal hemorrhage			
196	9 (3.5%)	134 (3.3%)	0.9881
Heart failure without cardiac catheter			
437	7 (2.8%)	113 (2.8%)	> 0.9999
Diabetes			
477	6 (2.4%)	109 (2.7%)	0.9036
Renal failure			
Allied health team in hospital			
Physiotherapy	221 (86.7%)	1340 (33.0%)	< 0.0001
Occupational therapy	178 (69.8%)	510 (12.6%)	< 0.0001
Speech language pathology	50 (19.6%)	149 (3.7%)	< 0.0001
Respiratory therapy	18 (7.1%)	175 (4.3%)	0.0575
Length of stay in days	30.85 (19.79–69.88)	3.95 (2.29–7.57)	< 0.0001
Median (IQR)			
ALC days	13.00		
Median (IQR)	(7.00–37.50)		
Hospital cost per patient ($)	22,459	5003	< 0.0001
Median (IQR)	(11,230-52,837)	(3627-8189)	
Complications in hospital			
Patients with any complication	65 (25.5%)	213 (5.3%)	< 0.0001
Delirium	12 (4.7%)	29 (0.7%)	< 0.0001
Aspiration	9 (3.5%)	16 (0.4%)	< 0.0001
Pulmonary embolism	0 (0%)	3 (0.1%)	> 0.9999
Congestive heart failure exacerbation	4 (1.6%)	47 (1.2%)	0.5424
Acute kidney injury	7 (2.8%)	57 (1.4%)	0.1013
Pressure ulcer	4 (1.6%)	3 (0.1%)	0.0004
Traumatic fractures	3 (1.2%)	2 (0.1%)	0.0019
Drug adverse effects	3 (1.2%)	10 (0.3%)	0.0376
Nosocomial infections	36 (14.1%)	76 (1.9%)	< 0.0001
Pneumonia	15 (5.9%)	24 (0.6%)	< 0.0001
Urinary tract infection	17 (6.7%)	34 (0.8%)	< 0.0001
*Clostridioides difficile* colitis	8 (3.1%)	9 (0.2%)	< 0.0001
Sepsis	0 (0%)	12 (0.3%)	> 0.9999
Death in hospital	36 (14.1%)	166 (4.1%)	
Mortality rate	0.25	0.66	
Deaths / 100 patient days			
Discharge destination (of those who were discharged alive)			< 0.0001
Acute care hospital	6 (2.7%)	51 (1.3%)	
Rehabilitation	71 (32.4%)	65 (1.7%)	
Home	14 (6.4%)	2462 (63.3%)	
Home with community agency support	52 (23.7%)	1015 (26.1%)	
Retirement home	18 (8.2%)	145 (3.7%)	
Nursing home	38 (17.4%)	21 (0.5%)	
Chronic care	15 (6.9%)	32 (0.8%)	
Other	5 (2.3%)	99 (2.5%)	
Readmission to hospital within 30 days (of those who were discharged alive)	26/219 (11.9%)	676/3890 (17.4%)	0.0440

### Follow-up in hospital

ALC patients had significant longer length of stay, higher hospital cost and more complications than non-ALC patients **(**Table 1**)**. Sensitivity analysis with pair matching based on propensity score showed similar results as the primary analysis without matching (Additional file 1: Text S1).

More ALC days was associated with higher rate of complications, nosocomial infections and mortality rate (Additional file 1: Table S2). Of the 36 ALC patients who died, 9 (25%) had malignancies and were pursuing palliative care. After reviewing each ALC case that died, we could not find any other obvious patterns in terms of demographics or other characteristics. Most ALC patients who were discharged alive did not return to their original home settings and were discharged to different settings with more support signifying higher care needs (Additional file 1: Table S3).

### Clinical prediction rule for ALC designation

The study population was randomized to 2155 and 2156 patients in the derivation and validation cohorts respectively. Using the derivation cohort, univariate analyses of potential predictors of ALC designation are listed in Additional file 1: Table S4. The final multivariate model of significant predictors for ALC designation is listed in Table 2. Using the validation cohort, the likelihood ratios applying a simplified clinical prediction rule using a point system where each significant risk factor in the multivariable model is given 1 point are listed in Table 3. The ROC curve for the clinical prediction rule based on score cutoff (Additional file 1: Figure S2) has an AUC of 0.85. Sensitivity analyses (including one in which calculation of the prediction rule score was weighted by coefficients in the logistic regression model and another one in which different time cut-off points were used for time sensitive variables) are shown in Additional file 1: Table S5 and S6.

**Table 2:** Final multivariable logistic regression model of risk factors for ALC designation

Significant risk factors	Odds ratio for ALC designation OR (95% CI)	*P*-value
Age > =80 years	2.80 (1.85–4.29)	< 0.0001
Female	1.52 (1.00–2.31)	0.0496
Charlson Comorbidity		
Dementia	3.40 (2.05–5.59)	< 0.0001
Diabetes with complications	1.61 (1.02–2.53)	0.0380
Allied health team in hospital		
Physiotherapy	3.28 (1.76–6.28)	0.0002
Occupational therapy	6.15 (3.83–10.16)	< 0.0001
Speech language pathology	2.80 (1.57–4.89)	0.0004

**Table 3:** Diagnostic properties of clinical prediction rule applied to validation cohort

Score	ALC patients / Patients with same number of points Positive predictive value	ALC patients (*N* = 124)	Non-ALC patients (*N* = 2032)	Likelihood ratio (95% CI)
Point system				
0 point	2 / 459 (0.4%)	2 (1.6%)	457 (22.5%)	0.07 (0.02–0.28)
1 point	11 / 742 (1.5%)	11 (8.9%)	731 (36.0%)	0.25 (0.14–0.44)
2 points	17 / 440 (3.9%)	17 (13.7%)	423 (20.8%)	0.66 (0.42–1.03)
3 points	26 / 314 (8.3%)	26 (21.0%)	288 (14.2%)	1.48 (1.03–2.12)
4 points	40 / 148 (27.0%)	40 (32.3%)	108 (5.3%)	6.07 (4.43–8.31)
5 points	23 / 45 (51.1%)	23 (18.6%)	22 (1.1%)	17.13 (9.83–29.86)
6 points	4 / 7 (57.1%)	4 (3.2%)	3 (0.2%)	21.85 (4.94–96.56)
7 points	1 / 1 (100%)	1 (0.8%)	0 (0%)	N/A
Risk category				
Low risk (0–1 points)	13 / 1201 (1.1%)	13 (10.5%)	1188 (58.5%)	0.18 (0.11–0.30)
Medium risk (2–3 points)	43 / 754 (5.7%)	43 (34.7%)	711 (35.0%)	0.99 (0.77–1.27)
High risk (> = 4 points)	68 / 201 (33.8%)	68 (54.8%)	133 (6.6%)	8.38 (6.66–10.54)

In the clinical prediction rule, score for each patient is calculated where each of the predictors in the multivariate model in Table 3 is worth 1 point (Age > =80 years = 1 point; female = 1 point; dementia = 1 point; diabetes with complications = 1 point; physiotherapy = 1 point; occupational therapy = 1 point; speech language pathologist = 1 point). N/A = not applicable as too few patients to accurately calculate likelihood ratios

## Discussion

In this retrospective cohort study, 6% of general internal medicine inpatients had delayed discharge as defined by ALC designation. Delayed discharge and consequent longer hospital stay were associated with significantly higher hospital costs and more complications in hospitals especially nosocomial infections. Unsurprisingly, these adverse effects increased as length of discharge delay increased. The most significant risk factors for delayed discharge included advanced age, female sex, diagnosis of dementia or diabetes with complication, and referrals to physiotherapy, occupational therapy and speech language pathology. A simple clinical prediction rule using a point system for each of these factors identified patients at heightened risk for delayed discharge. An easy mnemonic for the prediction rule is SAD PODS (S for Sex, A for Age, D for Dementia, P for Physiotherapy, O for Occupational therapy, D for Diabetes with complications and S for Speech language pathology).

These findings are consistent with prior studies. Similar to our study, patients whose discharge was delayed were found to have complications in hospital especially nosocomial infections [36]. The increased cost associated with delayed discharge is consistent with prior health economic studies. These studies attributed increased cost of delayed discharge to patients occupying beds, delays in admission due to bed occupancy, cost of nursing staff and administration costs [1]. Most of the economic evaluation studies focused on trauma, surgical and ICU patients [4, 18–21]. Some included both medical and surgical patients [37, 38]. Ours is the first study to estimate incremental hospital cost of delayed discharges for GIM inpatients, which complements the findings in the aforementioned patient populations. The components of our clinical prediction rule representing cognitive impairment, difficulty with mobility and dependency for activities of daily living were also significant predictors of delayed discharge in prior studies [39–42].

There are plausible mechanisms for how each component of our clinical prediction rule leads to delayed discharge (Additional file 1: Figure S3). Older age is associated with increasing comorbidities and functional decline. Female patients are more likely to be widowed and originate from retirement home signifying increased baseline support in our study (Additional file 1: Table S7). Dementia by definition is associated with functional decline and thus is associated with inability to perform instrumental or basic activities of daily living. Diabetes with complications affects multiple organs including retinopathy, neuropathy and diabetic foot ulcers, which limit mobility and impede function. Referrals to physiotherapy, occupational therapy and speech language pathologist suggest recognition by the medical team of the patient’s need for assistance with mobility, activities of daily living and swallowing. All of these risk factors indicate a need for more support and possible alternative disposition destination, which would require time and effort to set up and coordinate, thereby delaying discharge.

There were several strengths to this study. First, the large sample size with recruitment over a full year allowed for more precise estimates. To our knowledge, this is the largest study to date describing delayed discharge for GIM inpatients from a hospital perspective. Second, as per hospital and provincial policy, ALC designation was based on a standardized definition, prospectively applied for all GIM inpatients, and documented accurately on hospital records. Third, the higher hospital costs and complication rates were consistent in different analyses such as increase in rate with higher ALC days and propensity score matching for demographics, comorbidity and reason for admission. Fourth, the data collection and verification were rigorous, consistent and complete based on a standardized protocol by CIHI [24].

Several limitations merit emphasis. First, this study used data from a retrospective discharge database that was not designed for our specific research question. As a result, some variables were not available but would be informative for our research question. In particular, information on mobility, activities of daily living and swallowing on admission were unavailable in the database. However, use of more easily available hospital administrative data such as referral to allied health team members made the clinical prediction rule more practical to use from a hospital system perspective. It is possible for these time dependent variables to have reverse causality, where patients with longer hospital stay were more likely to have involvement of allied health team members. It should be noted that the involvement of allied health team members was usually early on within the first few days of admission (Additional file 1: Figure S1). We have also presented clinical prediction rules that can be calculated at 3, 5 and 7 days following hospital admission, which had similar diagnostic properties (Additional file 1: Table S6). Future studies should try to substitute these time dependent variables with assessments of mobility, activities of daily living and swallowing conducted at the time of admission.

Second, duration of follow-up for ALC and non-ALC patients was different. In this study, follow-up stopped at discharge. For non-ALC patients, follow-up stopped when their medical issues were resolved because they would be discharged at this point. In contrast, ALC patients had longer follow-up for additional hospital stays beyond resolution of their active issue, because they could not be discharged. More event outcomes such as in-hospital deaths and complications likely reflect (at least in part) the longer exposure times for the ALC patients. This was reflected in the unadjusted in-hospital mortality, where the ALC patients had a higher mortality but a lower mortality rate after adjusting for patient days. Due to this potential bias, our study only described the mortality rates. We did not perform any comparative statistics or draw any conclusions for mortality. For complications, we analyzed complications that could be attributable to hospitalization such that the risk would be less significant or non-existent following hospital discharge. For example, by definition, nosocomial infections occur only in hospital and thus were no longer applicable to patients who were discharged. Therefore, non-ALC patients who were discharged would not have any additional risk for nosocomial infections.

Third, the selection of the predictors to include in the clinical prediction rule was based in part on the clinical experience of our investigator team. However, we believe that the use of clinical judgment complements the statistical methods of stepwise regression by ensuring that the included risk factors both reflect significant associations and are clinically relevant.

Fourth, our study was based on a single academic acute care hospital, which limits the generalizability of results. The practice of caring for patients whose discharges were delayed differs across hospitals due to different hospital policies, physicians, allied health team composition and patient populations. For example, hospital services were publicly funded in this study. The length of delay to discharge may differ for other payment systems. Also, patient’s socioeconomic status such as income may be a more significant predictor of delayed discharge in other payment systems.

## Conclusions

Our study results suggest that delay in discharge may be a useful quality of care marker that needs to be measured by hospitals, because it is associated with higher hospital costs and complications. A simple clinical prediction rule can identify patients at high risk for delayed discharges. These patients can then be targeted for interventions to facilitate earlier discharges. Borghans et al. listed 50 such possible interventions at the hospital level [43]. On this list, possible evidence-based interventions included clinical pathways for specific patient groups, early rehabilitation on weekends and early anticipatory interdisciplinary discharge planning [43]. Interventions that minimize delays in discharge may decrease hospital costs and prevent hospital complications.

Future research should validate or improve upon our clinical prediction rule in identifying patients at high risk for delayed discharge and evaluate effectiveness of interventions targeted at this population in reducing delay in discharge and improving quality of care.

## Supplementary information

**Additional file 1:** **Table S1.** Complete Charlson Comorbidity Index and CMG for ALC and non-ALC patients**. Table S2.** Mortality and complication rate for different length of ALC days**. Table S3.** The original placement and discharge disposition for ALC patients who were discharged alive. **Table S4.** Univariate logistic regression model of potential predictors of ALC designation in the derivation cohort**. Table S5.** Diagnostic properties of clinical prediction rule applied to validation cohort using score weighted by coefficient of logistic regression model**. Table S6.** Diagnostic properties of clinical prediction rule applied to validation cohort using different time cut-off points**. Table S7.** Comparison of baseline characteristics between male and female sex**. Figure S1.** Time to allied health services**. Figure S2.** ROC curve of clinical prediction rule at different point cutoffs**. Figure S3.** Directed acyclic graph of proposed causal pathway of how SAD PODS risk factors lead to delayed discharge. **Text S1.** Propensity Score Analyses

## Data Availability

The datasets used and/or analyzed during the current study are available from the corresponding author on reasonable request.

## Abbreviations

ALC: Alternate level of care
GIM: General internal medicine
