# Supplementary material for: Risk factors, costs and complications of delayed hospital discharge from internal medicine wards at a Canadian academic medical centre: retrospective cohort study
Source: BMC Health Serv Res. 2019 Dec 4;19:935. doi: 10.1186/s12913-019-4760-3 (PMC6894295; doi:10.1186/s12913-019-4760-3)
Supplement: Supplementary file 1 — Additional file 1: Table S1. Complete Charlson Comorbidity Index and CMG for ALC and non-ALC patients. Table S2. Mortality and complication rate for different length of ALC days. Table S3. The original placement and discharge disposition for ALC patients who were discharged alive. Table S4. Univariate logistic regression model of potential predictors of ALC designation in the derivation cohort. Table S5. Diagnostic properties of clinical prediction rule applied to validation cohort using score weighted by coefficient of logistic regression model. Table S6. Diagnostic properties of clinical prediction rule applied to validation cohort using different time cut-off points. Table S7. Comparison of baseline characteristics between male and female sex. Figure S1. Time to allied health services. Figure S2. ROC curve of clinical prediction rule at different point cutoffs. Figure S3. Directed acyclic graph of proposed causal pathway of how SAD PODS risk factors lead to delayed discharge. Text S1. Propensity Score Analyses [file 12913_2019_4760_MOESM1_ESM.docx]

**SUPPLEMENTARY MATERIAL**

Appendix Table 1. Complete Charlson Comorbidity Index and CMG for ALC and non-ALC patients

Appendix Table 2. Mortality and complication rate for different length of ALC days

Appendix Table 3. The original placement and discharge disposition for ALC patients who were discharged alive

Appendix Table 4. Univariate logistic regression model of potential predictors of ALC designation in the derivation cohort

Appendix Table 5. Diagnostic properties of clinical prediction rule applied to validation cohort using score weighted by coefficient of logistic regression model

Appendix Table 6. Diagnostic properties of clinical prediction rule applied to validation cohort using different time cut-off points

Appendix Table 7. Comparison of baseline characteristics between male and female sex

Appendix Figure 1. Time to allied health services

Appendix Figure 2. ROC curve of clinical prediction rule at different point cutoffs

Appendix Figure 3. Directed acyclic graph of proposed causal pathway of how SAD PODS risk factors lead to delayed discharge

Appendix Text 1. Propensity Score Analyses

Appendix Table 1. Complete Charlson Comorbidity Index and CMG for ALC and non-ALC patients

|  | ALC patients  (N = 255) | Non-ALC patients  (N = 4056) | P-value |
| --- | --- | --- | --- |
| Charlson Comorbidity |  |  |  |
| Myocardial infarction | 7 (2.8%) | 64 (1.6%) | 0.2433 |
| Heart failure | 39 (15.3%) | 370 (9.1%) | 0.0016 |
| Peripheral vascular disease | 7 (2.8%) | 56 (1.4%) | 0.1357 |
| Cerebrovascular disease | 19 (7.5%) | 83 (2.1%) | <0.0001 |
| Dementia | 79 (31.0%) | 184 (4.5%) | <0.0001 |
| Chronic pulmonary disease | 35 (13.7%) | 749 (18.5%) | 0.0688 |
| Rheumatic disease | 3 (1.2%) | 71 (1.8%) | 0.8012 |
| Peptic ulcer disease | 5 (2.0%) | 83 (2.1%) | >0.9999 |
| Mild liver disease | 8 (3.1%) | 177 (4.4%) | 0.4364 |
| Moderate or severe liver disease | 8 (3.1%) | 111 (2.7%) | 0.8558 |
| Diabetes mellitus without end  organ damage | 44 (17.3%) | 543 (13.4%) | 0.0984 |
| Diabetes mellitus with end organ  damage | 75 (29.4%) | 790 (19.5%) | 0.0002 |
| Hemiplegia or paraplegia | 3 (1.2%) | 22 (0.5%) | 0.1813 |
| Moderate or severe renal disease | 13 (5.1%) | 210 (5.2%) | >0.9999 |
| Solid tumor, leukemia or lymphoma | 16 (6.3%) | 321 (7.9%) | 0.4089 |
| Metastatic solid tumor | 17 (6.7%) | 258 (6.4%) | 0.9508 |
| HIV / AIDS | 1 (0.4%) | 10 (0.3%) | 0.4891 |
| Top 52 CMG |  |  |  |
| 139  Chronic obstructive pulmonary disease | 11 (4.3%) | 430 (10.6%) | 0.0019 |
| 138  Viral / unspecified pneumonia | 15 (5.9%) | 232 (5.7%) | >0.9999 |
| 487  Lower urinary tract infection | 17 (6.7%) | 173 (4.3%) | 0.0980 |
| 254  Gastrointestinal hemorrhage | 4 (1.6%) | 145 (3.6%) | 0.1090 |
| 196  Heart failure without cardiac catheter | 9 (3.5%) | 134 (3.3%) | 0.9881 |
| 437  Diabetes | 7 (2.8%) | 113 (2.8%) | >0.9999 |
| 477  Renal failure | 6 (2.4%) | 109 (2.7%) | 0.9036 |
| 249  Non-severe enteritis | 1 (0.4%) | 104 (2.6%) | 0.0203 |
| 257  Symptom/sign of digestive system | 2 (0.8%) | 88 (2.2%) | 0.1734 |
| 436  Disorder of fluid/electrolyte balance | 0 (0%) | 84 (2.1%) | 0.0092 |
| 778  Poisoning / toxic effect of drug | 2 (0.8%) | 76 (1.9%) | 0.3267 |
| 132  Malignant neoplasm of respiratory system | 4 (1.6%) | 68 (1.7%) | >0.9999 |
| 405  Cellulitis | 2 (0.8%) | 69 (1.7%) | 0.4406 |
| 285  Cirrhosis / alcoholic hepatitis | 6 (2.4%) | 63 (1.6%) | 0.4655 |
| 811  General symptom/sign | 12 (4.7%) | 56 (1.4%) | 0.0001 |
| 142  Other lung disease | 6 (2.4%) | 59 (1.5%) | 0.3805 |
| 231  Minor upper gastrointestinal intervention | 1 (0.4%) | 64 (1.6%) | 0.1822 |
| 287  Disorder of pancreas except malignancy | 1 (0.4%) | 63 (1.6%) | 0.1815 |
| 654  Other/unspecified septicemia | 4 (1.6%) | 60 (1.5%) | 0.7899 |
| 671  Organic mental disorder | 14 (5.5%) | 48 (1.2%) | <0.0001 |
| 248  Severe enteritis | 1 (0.4%) | 60 (1.5%) | 0.2651 |
| 135  Aspiration pneumonia | 8 (3.1%) | 50 (1.2%) | 0.0226 |
| 660  Other infectious / parasitic disease | 4 (1.6%) | 53 (1.3%) | 0.5784 |
| 200  Pulmonary embolism | 1 (0.4%) | 51 (1.3%) | 0.3691 |
| 209  Other / miscellaneous cardiac disorder | 2 (0.8%) | 50 (1.2%) | 0.7681 |
| 232  Minor lower gastrointestinal intervention | 1 (0.4%) | 50 (1.2%) | 0.3672 |
| 147  Asthma | 0 (0%) | 48 (1.2%) | 0.1143 |
| 149  Symptom/sign of respiratory system | 0 (0%) | 44 (1.1%) | 0.1104 |
| 258  Other gastrointestinal disorder | 1 (0.4%) | 41 (1.0%) | 0.5147 |
| 708  Substance abuse with other state | 2 (0.8%) | 40 (1.0%) | >0.9999 |
| 488  Upper urinary tract infection | 2 (0.8%) | 39 (1.0%) | >0.9999 |
| 253  Inflammatory bowel disease | 0 (0%) | 38 (0.9%) | 0.2274 |
| 250  Digestive malignancy | 3 (1.2%) | 34 (0.8%) | 0.4796 |
| 438  Dehydration | 3 (1.2%) | 33 (0.8%) | 0.4697 |
| 97  Influenza/acute upper respiratory  Infection | 0 (0%) | 33 (0.8%) | 0.2605 |
| 130  Respiratory failure | 6 (2.4%) | 26 (0.6%) | 0.0067 |
| 202  Arrhythmia without cardiac catheter | 3 (1.2%) | 29 (0.7%) | 0.4343 |
| 363  Other soft tissue disorder | 2 (0.8%) | 29 (0.7%) | 0.6054 |
| 284  Hepatobiliary / pancreatic malignancy | 3 (1.2%) | 25 (0.6%) | 0.2280 |
| 670  Dementia | 15 (5.9%) | 12 (0.3%) | <0.0001 |
| 662  Fever | 0 (0%) | 26 (0.6%) | 0.4005 |
| 37  Other dysfunction of central nervous  system | 1 (0.4%) | 24 (0.6%) | >0.9999 |
| 256   \| Esophagitis/gastritis/miscellaneous digestive disease \| \| --- \| | 0 (0%) | 25 (0.6%) | 0.3981 |
| 407  Other disease / disorder of skin /  subcutaneous tissue | 0 (0%) | 25 (0.6%) | 0.3981 |
| 206  Benign hypertension | 0 (0%) | 24 (0.6%) | 0.3969 |
| 635  Other anemia | 2 (0.8%) | 22 (0.5%) | 0.6502 |
| 143  Disease of pleura | 0 (0%) | 23 (0.6%) | 0.3969 |
| 286  Liver disease except cirrhosis /  Malignancy | 0 (0%) | 23 (0.6%) | 0.3969 |
| 435  Disorder of metabolism | 2 (0.8%) | 21 (0.5%) | 0.6436 |
| 370  Inflammatory and reactive arthropathy | 0 (0%) | 22 (0.5%) | 0.6377 |
| 480  Kidney disease | 1 (0.4%) | 21 (0.5%) | >0.9999 |
| 698  Psychoactive substance use, acute  intoxication | 0 (0%) | 22 (0.5%) | 0.6377 |

Appendix Table 2. Mortality and complication rate for different length of ALC days

|  | 0-7 ALC days  (N=84) | 8-14 ALC days  (N=53) | 15-35 ALC days  (N=52) | >35 ALC days  (N=66) |
| --- | --- | --- | --- | --- |
| Any complications | 13 (15.5%) | 9 (17.0%) | 14 (26.9%) | 29 (43.9%) |
| Nosocomial infections | 7 (8.3%) | 4 (7.6%) | 5 (9.6%) | 20 (30.3%) |
| Mortality | 9 (10.7%) | 5 (9.4%) | 10 (19.2%) | 12 (18.2%) |

Appendix Table 3. The original placement and discharge disposition for ALC patients who were discharged alive

|  | From home  (N=166) | From retirement home  (N=40) | From other  (N=13) |
| --- | --- | --- | --- |
| To acute care hospital | 5 (3.1%) | 0 (0%) | 1 (7.7%) |
| To rehabilitation | 54 (32.5%) | 15 (37.5%) | 2 (15.4%) |
| To home | 11 (6.7%) | 1 (2.5%) | 2 (15.4%) |
| To home with community  agency support | 43 (25.9%) | 8 (20.0%) | 1 (7.7%) |
| To retirement home | 11 (6.6%) | 6 (15.0%) | 1 (7.7%) |
| To nursing home | 26 (15.7%) | 8 (20.0%) | 4 (30.8%) |
| To chronic care | 12 (7.2%) | 2 (5.0%) | 1 (7.7%) |
| To other | 4 (2.4%) | 0 (0%) | 1 (7.7%) |

The 5 ALC patients discharged to other included 4 patients to psychiatric facilities and 1 patient to an unclassified health facility

Appendix Table 4. Univariate logistic regression model of potential predictors of ALC designation in the derivation cohort

| Significant risk factors | Odds ratio for ALC designation  OR (95% CI) | P-value |
| --- | --- | --- |
| Age per year | 1.06 (1.05-1.08) | <0.0001 |
| Age category |  |  |
| <80 years | Reference |  |
| >=80 years | 5.49 (3.81-7.99) | <0.0001 |
| Male | 0.76 (0.53-1.09) | 0.1330 |
| Median yearly income based on postal code per $ | 1.00002 (0.99998-1.00007) | 0.3340 |
| Marital status |  |  |
| Currently married | Reference |  |
| Widowed | 2.33 (1.53-3.53) | <0.0001 |
| Other | 0.75 (0.48-1.17) | 0.2150 |
| Admitted from |  |  |
| Home | Reference |  |
| Retirement home | 3.24 (1.86-5.39) | <0.0001 |
| Other | 2.60 (1.35-4.66) | 0.0023 |
| Charlson Comorbidity Index |  |  |
| 0 | Reference |  |
| 1 | 1.26 (0.73-2.18) | 0.3960 |
| >=2 | 1.95 (1.27-3.07) | 0.0029 |
| Charlson Comorbidity |  |  |
| Myocardial infarction | 0.83 (0.13-2.76) | 0.8020 |
| Heart failure | 1.66 (0.98-2.68) | 0.0457 |
| Peripheral vascular disease | 1.67 (0.40-4.80) | 0.4030 |
| Cerebrovascular disease | 4.06 (2.02-7.60) | <0.0001 |
| Dementia | 8.30 (5.36-12.69) | <0.0001 |
| Chronic pulmonary disease | 0.87 (0.52-1.38) | 0.5720 |
| Rheumatic disease | 0.97 (0.15-3.23) | 0.9610 |
| Peptic ulcer disease | 1.45 (0.43-3.65) | 0.4830 |
| Mild liver disease | 0.81 (0.28-1.85) | 0.6620 |
| Moderate or severe liver disease | 1.11 (0.33-2.75) | 0.8470 |
| Diabetes mellitus without end  organ damage | 1.31 (0.80-2.07) | 0.2640 |
| Diabetes mellitus with end organ  damage | 1.85 (1.25-2.70) | 0.0018 |
| Hemiplegia or paraplegia | 6.75 (1.44-24.60) | 0.0061 |
| Moderate or severe renal disease | 0.98 (0.41-2.01) | 0.9640 |
| Solid tumor, leukemia or lymphoma | 0.46 (0.16-1.02) | 0.0902 |
| Metastatic solid tumor | 1.12 (0.49-2.21) | 0.7620 |
| HIV / AIDS | 2.22 (0.12-12.59) | 0.4580 |
| Allied health services in hospital |  |  |
| Physiotherapy | 13.73 (8.41-23.86) | <0.0001 |
| Occupational therapy | 17.47 (11.78-26.40) | <0.0001 |
| Speech language pathologist | 7.02 (4.23-11.36) | <0.0001 |
| Respiratory therapist | 2.07 (1.02-3.81) | 0.0297 |
| Top 52 CMG |  |  |
| 139  Chronic obstructive pulmonary disease | 0.41 (0.16-0.86) | 0.0336 |
| 138  Viral / unspecified pneumonia | 1.29 (0.62-2.40) | 0.4590 |
| 487  Lower urinary tract infection | 2.09 (1.03-3.86) | 0.0272 |
| 254  Gastrointestinal hemorrhage | <0.01 (0-19.03) | 0.9750 |
| 196  Heart failure without cardiac catheter | 0.66 (0.16-1.82) | 0.4930 |
| 437  Diabetes | 1.09 (0.33-2.70) | 0.8740 |
| 477  Renal failure | 0.47 (0.08-1.54) | 0.3030 |
| 249  Non-severe enteritis | 0.31 (0.02-1.43) | 0.2480 |
| 257  Symptom/sign of digestive system | <0.01 (0-8.06) | 0.9700 |
| 436  Disorder of fluid/electrolyte balance | <0.01 (0->1000) | 0.9790 |
| 778  Poisoning / toxic effect of drug | 0.34 (0.02-1.57) | 0.2860 |
| 132  Malignant neoplasm of respiratory system | 0.42 (0.02-1.99) | 0.4000 |
| 405  Cellulitis | 0.48 (0.03-2.25) | 0.4700 |
| 285  Cirrhosis / alcoholic hepatitis | 1.41 (0.34-4.01) | 0.5700 |
| 811  General symptom/sign | 3.69 (1.35-8.56) | 0.0047 |
| 142  Other lung disease | 0.51 (0.03-2.41) | 0.5110 |
| 231  Minor upper gastrointestinal intervention | 0.45 (0.03-2.11) | 0.4330 |
| 287  Disorder of pancreas except malignancy | 0.41 (0.02-1.93) | 0.3850 |
| 654  Other/unspecified septicemia | 1.33 (0.32-3.76) | 0.6380 |
| 671  Organic mental disorder | 3.45 (1.14-8.54) | 0.0136 |
| 248  Severe enteritis | 0.59 (0.03-2.81) | 0.6070 |
| 135  Aspiration pneumonia | 2.47 (0.58-7.38) | 0.1490 |
| 660  Other infectious / parasitic disease | 1.80 (0.43-5.20) | 0.3400 |
| 200  Pulmonary embolism | 0.51 (0.03-2.41) | 0.5110 |
| 209  Other / miscellaneous cardiac disorder | 1.19 (0.19-4.04) | 0.8130 |
| 232  Minor lower gastrointestinal intervention | 0.55 (0.03-2.60) | 0.5560 |
| 147  Asthma | N/A | N/A |
| 149  Symptom/sign of respiratory system | N/A | N/A |
| 258  Other gastrointestinal disorder | N/A | N/A |
| 708  Substance abuse with other state | 0.91 (0.05-4.47) | 0.9260 |
| 488  Upper urinary tract infection | N/A | N/A |
| 253  Inflammatory bowel disease | N/A | N/A |
| 250  Digestive malignancy | N/A | N/A |
| 438  Dehydration | 1.73 (0.27-6.07) | 0.4660 |
| 97  Influenza/acute upper respiratory  Infection | N/A | N/A |
| 130  Respiratory failure | 5.70 (1.82-15.15) | 0.0010 |
| 202  Arrhythmia without cardiac catheter | 2.94 (0.68-8.97) | 0.0897 |
| 363  Other soft tissue disorder | 1.29 (0.07-6.62) | 0.8080 |
| 284  Hepatobiliary / pancreatic malignancy | 2.60 (0.40-9.66) | 0.2140 |
| 670  Dementia | 12.10 (3.93-35.31) | <0.0001 |
| 662  Fever | N/A | N/A |
| 37  Other dysfunction of central nervous  system | N/A | N/A |
| 256   \| Esophagitis/gastritis/miscellaneous digestive disease \| \| --- \| | N/A | N/A |
| 407  Other disease / disorder of skin /  subcutaneous tissue | N/A | N/A |
| 206   \| Benign hypertension \| \| --- \| | N/A | N/A |
| 635  Other anemia | 2.59 (0.14-15.30) | 0.3800 |
| 143  Disease of pleura | N/A | N/A |
| 286  Liver disease except cirrhosis /  Malignancy | N/A | N/A |
| 435  Disorder of metabolism | 1.19 (0.07-6.04) | 0.8670 |
| 370  Inflammatory and reactive arthropathy | N/A | N/A |
| 480  Kidney disease | N/A | N/A |
| 698  Psychoactive substance use, acute  intoxication | N/A | N/A |

N/A = not applicable for which the 2x2 table have too few patients in one cell to calculate accurately an odds ratio

Appendix Table 5. Diagnostic properties of clinical prediction rule applied to validation cohort using score weighted by coefficient of logistic regression model

| Score | ALC patients / Patients with same number of points  Positive predictive value | ALC patients  (N=124) | Non-ALC patients  (N=2032) | Likelihood ratio  (95% CI) |
| --- | --- | --- | --- | --- |
| Point system | | | | |
| 0 point | 2 / 459 (0.4%) | 2 (1.6%) | 457 (22.5%) | 0.07 (0.02-0.28) |
| 1 point | 4 / 536 (0.8%) | 4 (3.2%) | 532 (26.2%) | 0.12 (0.05-0.32) |
| 2 points | 1 / 158 (0.6%) | 1 (0.8%) | 157 (7.7%) | 0.10 (0.02-0.74) |
| 3 points | 10 / 241 (4.2%) | 10 (8.1%) | 231 (11.4%) | 0.71 (0.39-1.30) |
| 4 points | 3 / 154 (2.0%) | 3 (2.4%) | 151 (7.4%) | 0.33 (0.11-1.01) |
| 5 points | 5 / 114 (4.4%) | 5 (4.0%) | 109 (5.4%) | 0.75 (0.31-1.81) |
| 6 points | 7 / 118 (5.9%) | 7 (5.7%) | 111 (5.5%) | 1.03 (0.49-2.17) |
| 7 points | 7 / 65 (10.8%) | 7 (5.7%) | 58 (2.9%) | 1.98 (0.92-4.24) |
| 8 points | 18 / 92 (19.6%) | 18 (14.5%) | 74 (3.6%) | 3.99 (2.46-6.46) |
| 9 points | 10 / 76 (13.2%) | 10 (8.1%) | 66 (3.3%) | 2.48 (1.31-4.71) |
| 10 points | 18 / 70 (25.7%) | 18 (14.5%) | 52 (2.6%) | 5.67 (3.43-9.39) |
| 11 points | 9 / 21 (42.9%) | 9 (7.3%) | 12 (0.6%) | 12.29 (5.28-28.61) |
| 12 points | 12 / 21 (57.1%) | 12 (9.7%) | 9 (0.4%) | 21.85 (9.39-50.87) |
| 13 points | 8 / 16 (50.0%) | 8 (6.5%) | 8 (0.4%) | 16.39 (6.26-42.93) |
| 14 points | 6 / 9 (66.7%) | 6 (4.8%) | 3 (0.2%) | 32.77 (8.30-129.50) |
| 15 points | 3 / 5 (60.0%) | 3 (2.4%) | 2 (0.1%) | 24.58 (4.14-145.76) |
| 16 points | 1 / 1 (100.0%) | 1 (0.8%) | 0 (0%) | N/A |

In the clinical prediction rule, score for each patient is calculated where each of the predictors in the multivariate model in table 3 is worth based on ratio to the lowest coefficient in the logistic regression model (Age >=80 years = 2 points; female = 1 point; dementia = 3 points; diabetes with complications = 1 point; physiotherapy = 3 points; occupational therapy = 4 points; speech language pathologist = 2 points). N/A = not applicable as too few patients to accurately calculate likelihood ratios. The AUC was 0.87.

Appendix Table 6. Diagnostic properties of clinical prediction rule applied to validation cohort using different time cut-off points

| Score | ALC patients / Patients with same number of points  Positive predictive value | ALC patients  (N=124) | Non-ALC patients  (N=2032) | Likelihood ratio  (95% CI) |
| --- | --- | --- | --- | --- |
| **At 3 days** | | | | |
| 0 point | 11 / 535 (2.1%) | 11 (8.9%) | 524 (25.8%) | 0.34 (0.20-0.61) |
| 1 point | 18 / 836 (2.2%) | 18 (14.5%) | 818 (40.3%) | 0.36 (0.23-0.56) |
| 2 points | 38 / 475 (8.0%) | 38 (30.7%) | 437 (21.5%) | 1.43 (1.08-1.88) |
| 3 points | 34 / 239 (14.2%) | 34 (27.4%) | 205 (10.1%) | 2.72 (1.99-3.72) |
| 4 points | 13 / 54 (24.1%) | 13 (10.5%) | 41 (2.0%) | 5.20 (2.86-9.44) |
| 5 points | 7 / 11 (63.6%) | 7 (5.7%) | 4 (0.2%) | 28.68 (8.51-96.66) |
| 6 points | 3 / 6 (50.0%) | 3 (2.4%) | 3 (0.2%) | 16.39 (3.34-80.36) |
| **At 5 days** | | | | |
| 0 point | 7 / 505 (1.4%) | 7 (5.7%) | 498 (24.5%) | 0.23 (0.11-0.48) |
| 1 point | 17 / 788 (2.2%) | 17 (13.7%) | 771 (37.9%) | 0.36 (0.23-0.56) |
| 2 points | 28 / 472 (5.9%) | 28 (22.6%) | 444 (21.9%) | 1.03 (0.74-1.45) |
| 3 points | 39 / 276 (14.1%) | 39 (31.5%) | 237 (11.7%) | 2.70 (2.03-3.59) |
| 4 points | 21 / 90 (23.3%) | 21 (16.9%) | 69 (3.4%) | 4.99 (3.17-7.85) |
| 5 points | 9 / 19 (47.3%) | 9 (7.3%) | 10 (0.5%) | 14.75 (6.10-35.63) |
| 6 points | 3 / 6 (50.0%) | 3 (2.4%) | 3 (0.2%) | 16.39 (3.34-80.36) |
| **At 7 days** | | | | |
| 0 point | 6 / 488 (1.2%) | 6 (4.8%) | 482 (23.7%) | 0.20 (0.09-0.45) |
| 1 point | 15 / 774 (1.9%) | 15 (12.1%) | 759 (37.4%) | 0.32 (0.20-0.52) |
| 2 points | 27 / 465 (5.8%) | 27 (21.8%) | 438 (21.6%) | 1.01 (0.72-1.43) |
| 3 points | 37 / 294 (12.6%) | 37 (29.8%) | 257 (12.7%) | 2.36 (1.76-3.16) |
| 4 points | 22 / 99 (22.2%) | 22 (17.7%) | 77 (3.8%) | 4.68 (3.02-7.25) |
| 5 points | 14 / 30 (46.7%) | 14 (11.3%) | 16 (0.8%) | 14.34 (7.16-28.70) |
| 6 points | 3 / 6 (50.0%) | 3 (2.4%) | 3 (0.2%) | 16.39 (3.34-80.36) |

AUC for cut-off at 3 days, 5 days and 7 days are 0.75, 0.78 and 0.79 respectively.

Appendix Table 7. Comparison of baseline characteristics between male and female sex

|  | Females (N=2151) | Males (N=2160) | P-value |
| --- | --- | --- | --- |
| Age median (IQR) | 70.0 (55.0-81.0) | 69.0 (56.0-80.0) | 0.4245 |
| Age by category |  |  |  |
| <80 years | 1562 (72.6%) | 1600 (74.1%) | 0.2950 |
| >=80 years | 589 (27.4%) | 560 (25.9%) |  |
| Median yearly income based on postal code per $ | 35,277  (31,883-36,685) | 32,277  (32,410-36,685) | 0.2154 |
| Marital status |  |  |  |
| Currently married | 811 (37.7%) | 1106 (51.2%) | <0.0001 |
| Widowed | 582 (27.1%) | 212 (9.8%) |  |
| Other | 758 (35.2%) | 842 (39.0%) |  |
| Admitted from |  |  |  |
| Home | 1894 (88.1%) | 1932 (89.4%) | <0.0001 |
| Retirement home | 204 (9.5%) | 92 (4.3%) |  |
| Other | 53 (2.5%) | 136 (6.3%) |  |
| Charlson Comorbidity Index |  |  |  |
| 0 | 760 (35.3%) | 612 (28.3%) | <0.0001 |
| 1 | 549 (25.5%) | 449 (20.8%) |  |
| >=2 | 842 (39.1%) | 1099 (50.9%) |  |

Appendix Figure 1. Time to allied health services


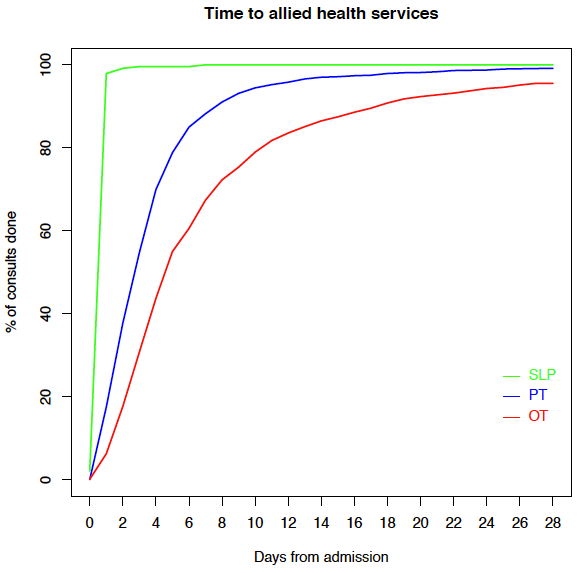


The timing refers to completion of allied health assessment. Typically, the referral was made 24-48 hours prior to this. Therefore, the timing displayed over-estimates the time to allied health services involvement.

Appendix Figure 2. ROC curve of clinical prediction rule at different point cutoffs


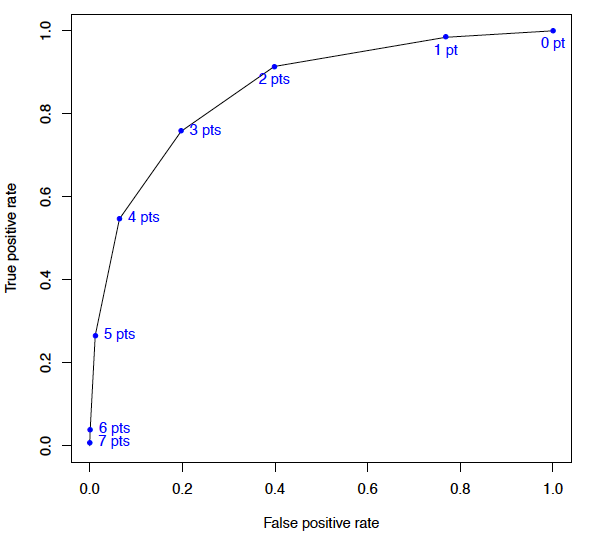


Appendix Figure 3. Directed acyclic graph of proposed causal pathway of how SAD PODS risk factors lead to delayed discharge


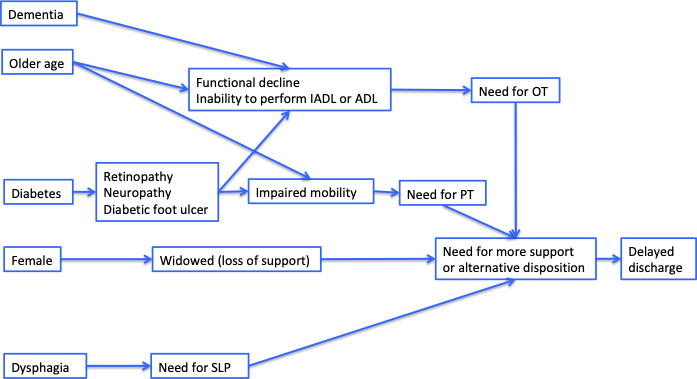


Appendix Text 1. Propensity Score Analyses

**Methods**

A propensity score for ALC designation was estimated using a multivariable logistic regression model.^1^ The following covariates were included in the model: age, sex, original placement, Charlson comorbidity, CCI score and CMG. ALC patients were matched in a 1:1 ratio to non-ALC patients using nearest neighbor matching with a specified caliper width of 0.2 times SD of the logit of the propensity score.^2^ Covariate balance between the two groups was assessed by standardized difference.^3^

Matching was done using the package matchit (Ho MatchIt package).^4^

**Results**

Based on propensity score, 243 of the 255 ALC patients were matched to 243 non-ALC patients. The propensity matched cohorts were similar with respect to variables matched by propensity score (Table 1).

In the propensity matched cohorts, ALC patients had longer length of stay, higher cost and higher number of complications (Table 2). These findings were consistent with the primary analysis without matching.

Table 1. Complete Charlson Comorbidity Index and CMG for propensity matched ALC and non-ALC patients

|  | ALC patients  (N = 243) | Non-ALC patients  (N = 243) | SDM |
| --- | --- | --- | --- |
| Age median (IQR) | 81.0 (72.0-88.0) | 81.0 (72.0-87.0) | 0.0401 |
| Male | 102 (42.0%) | 104 (42.8%) | 0.0167 |
| Median yearly income based on postal code median (IQR) | 35,277  (32,410-36,685) | 35,277  (31,883-36,685) | 0.0445 |
| Marital status |  |  |  |
| Currently married | 95 (39.1%) | 99 (40.7%) | 0.0336 |
| Widowed | 82 (33.7%) | 77 (31.7%) | 0.0439 |
| Other | 66 (27.2%) | 67 (27.6%) | 0.0092 |
| Admitted from |  |  |  |
| Home | 180 (74.1%) | 179 (73.7%) | 0.0094 |
| Retirement home | 48 (19.8%) | 46 (18.9%) | 0.0208 |
| Other | 15 (6.2%) | 18 (7.4%) | 0.0491 |
| Charlson Comorbidity Index |  |  |  |
| 0 | 48 (19.8%) | 37 (15.2%) | 0.1194 |
| 1 | 57 (23.5%) | 72 (29.6%) | 0.1401 |
| >=2 | 138 (56.8%) | 134 (55.1%) | 0.0332 |
| Charlson Comorbidity |  |  |  |
| Myocardial infarction | 7 (2.9%) | 6 (2.5%) | 0.0255 |
| Heart failure | 35 (14.4%) | 34 (14.0%) | 0.0118 |
| Peripheral vascular disease | 7 (2.9%) | 9 (3.7%) | 0.0461 |
| Cerebrovascular disease | 16 (6.6%) | 17 (7.0%) | 0.0164 |
| Dementia | 68 (28.0%) | 63 (25.9%) | 0.0464 |
| Chronic pulmonary disease | 33 (13.6%) | 34 (14.0%) | 0.0119 |
| Rheumatic disease | 3 (1.2%) | 5 (2.1%) | 0.0647 |
| Peptic ulcer disease | 4 (1.7%) | 7 (2.9%) | 0.0831 |
| Mild liver disease | 8 (3.3%) | 8 (3.3%) | 0 |
| Moderate or severe liver disease | 7 (2.9%) | 7 (2.9%) | 0 |
| Diabetes mellitus without end  organ damage | 39 (16.1%) | 45 (18.5%) | 0.0653 |
| Diabetes mellitus with end organ  damage | 69 (28.4%) | 67 (27.6%) | 0.0183 |
| Hemiplegia or paraplegia | 3 (1.2%) | 2 (0.8%) | 0.0408 |
| Moderate or severe renal disease | 12 (4.9%) | 9 (3.7%) | 0.0607 |
| Solid tumor, leukemia or lymphoma | 16 (6.6%) | 18 (7.4%) | 0.0323 |
| Metastatic solid tumor | 17 (7.0%) | 18 (7.4%) | 0.0159 |
| HIV / AIDS | 1 (0.4%) | 2 (0.8%) | 0.0526 |
| Top 52 CMG |  |  |  |
| 139  Chronic obstructive pulmonary disease | 11 (4.5%) | 11 (4.5%) | 0 |
| 138  Viral / unspecified pneumonia | 15 (6.2%) | 12 (4.9%) | 0.0539 |
| 487  Lower urinary tract infection | 17 (7.0%) | 17 (7.0%) | 0 |
| 254  Gastrointestinal hemorrhage | 4 (1.7%) | 6 (2.5%) | 0.0580 |
| 196  Heart failure without cardiac catheter | 8 (3.3%) | 6 (2.5%) | 0.0492 |
| 437  Diabetes | 7 (2.9%) | 8 (3.3%) | 0.0238 |
| 477  Renal failure | 6 (2.5%) | 5 (2.1%) | 0.0277 |
| 249  Non-severe enteritis | 1 (0.4%) | 1 (0.4%) | 0 |
| 257  Symptom/sign of digestive system | 2 (0.8%) | 1 (0.4%) | 0.0526 |
| 436  Disorder of fluid/electrolyte balance | 0 (0%) | 0 (0%) | 0 |
| 778  Poisoning / toxic effect of drug | 2 (0.8%) | 2 (0.8%) | 0 |
| 132  Malignant neoplasm of respiratory system | 4 (1.7%) | 2 (0.8%) | 0.0746 |
| 405  Cellulitis | 2 (0.8%) | 1 (0.4%) | 0.0526 |
| 285  Cirrhosis / alcoholic hepatitis | 6 (2.5%) | 4 (1.7%) | 0.0580 |
| 811  General symptom/sign | 12 (4.9%) | 12 (4.9%) | 0 |
| 142  Other lung disease | 6 (2.5%) | 5 (2.1%) | 0.0277 |
| 231  Minor upper gastrointestinal intervention | 1 (0.4%) | 0 (0%) | 0.0909 |
| 287  Disorder of pancreas except malignancy | 1 (0.4%) | 1 (0.4%) | 0 |
| 654  Other/unspecified septicemia | 4 (1.7%) | 4 (1.7%) | 0 |
| 671  Organic mental disorder | 12 (4.9%) | 13 (5.4%) | 0.0186 |
| 248  Severe enteritis | 1 (0.4%) | 1 (0.4%) | 0 |
| 135  Aspiration pneumonia | 8 (3.3%) | 11 (4.5%) | 0.0637 |
| 660  Other infectious / parasitic disease | 4 (1.7%) | 2 (0.8%) | 0.0746 |
| 200  Pulmonary embolism | 1 (0.4%) | 1 (0.4%) | 0 |
| 209  Other / miscellaneous cardiac disorder | 2 (0.8%) | 1 (0.4%) | 0.0526 |
| 232  Minor lower gastrointestinal intervention | 1 (0.4%) | 2 (0.8%) | 0.0526 |
| 147  Asthma | 0 (0%) | 0 (0%) | 0 |
| 149  Symptom/sign of respiratory system | 0 (0%) | 0 (0%) | 0 |
| 258  Other gastrointestinal disorder | 1 (0.4%) | 1 (0.4%) | 0 |
| 708  Substance abuse with other state | 2 (0.8%) | 2 (0.8%) | 0 |
| 488  Upper urinary tract infection | 2 (0.8%) | 4 (1.7%) | 0.0746 |
| 253  Inflammatory bowel disease | 0 (0%) | 0 (0%) | 0 |
| 250  Digestive malignancy | 3 (1.2%) | 3 (1.2%) | 0 |
| 438  Dehydration | 3 (1.2%) | 2 (0.8%) | 0.0408 |
| 97  Influenza/acute upper respiratory  Infection | 0 (0%) | 0 (0%) | 0 |
| 130  Respiratory failure | 6 (2.5%) | 8 (3.3%) | 0.0492 |
| 202  Arrhythmia without cardiac catheter | 3 (1.2%) | 1 (0.4%) | 0.0912 |
| 363  Other soft tissue disorder | 2 (0.8%) | 2 (0.8%) | 0 |
| 284  Hepatobiliary / pancreatic malignancy | 3 (1.2%) | 3 (1.2%) | 0 |
| 670  Dementia | 9 (3.7%) | 6 (2.5%) | 0.0714 |
| 662  Fever | 0 (0%) | 0 (0%) | 0 |
| 37  Other dysfunction of central nervous  system | 1 (0.4%) | 2 (0.8%) | 0.0526 |
| 256   \| Esophagitis/gastritis/miscellaneous digestive disease \| \| --- \| | 0 (0%) | 1 (0.4%) | 0.0909 |
| 407  Other disease / disorder of skin /  subcutaneous tissue | 0 (0%) | 0 (0%) | 0 |
| 206  Benign hypertension | 0 (0%) | 1 (0.4%) | 0.0909 |
| 635  Other anemia | 2 (0.8%) | 3 (1.2%) | 0.0408 |
| 143  Disease of pleura | 0 (0%) | 0 (0%) | 0 |
| 286  Liver disease except cirrhosis /  Malignancy | 0 (0%) | 0 (0%) | 0 |
| 435  Disorder of metabolism | 2 (0.8%) | 2 (0.8%) | 0 |
| 370  Inflammatory and reactive arthropathy | 0 (0%) | 0 (0%) | 0 |
| 480  Kidney disease | 1 (0.4%) | 1 (0.4%) | 0 |
| 698  Psychoactive substance use, acute  intoxication | 0 (0%) | 0 (0%) | 0 |
| Allied health referrals in hospital |  |  |  |
| Physiotherapy | 211 (86.8%) | 123 (50.6%) | 0.8485 |
| Occupational therapy | 170 (70.0%) | 67 (27.6%) | 0.9363 |
| Speech language pathologist | 46 (18.9%) | 16 (6.6%) | 0.3766 |
| Respiratory therapist | 17 (7.0%) | 8 (3.3%) | 0.1683 |

Table 2. Outcome of propensity score matched ALC and non-ALC patients

|  | ALC patients  (N = 243) | Non-ALC patients  (N = 243) | P-value |
| --- | --- | --- | --- |
| Length of stay in days  Median (IQR) | 30.3 (19.5-66.4) | 4.9 (2.6-10.8) | <0.0001 |
| Hospital cost ($)  Median (IQR) | 21,513  (10,994-47,351) | 6,423  (3,889-10,600) | <0.0001 |
| Complications in hospital |  |  |  |
| Any complications | 59 (24.3%) | 24 (9.9%) | <0.0001 |
| Delirium | 10 (4.1%) | 2 (0.8%) | 0.0363 |
| Aspiration | 7 (2.9%) | 5 (2.1%) | 0.7716 |
| Pulmonary embolism | 0 (0%) | 0 (0%) | N/A |
| Congestive heart failure exacerbation | 3 (1.2%) | 5 (2.1%) | 0.7243 |
| Acute kidney injury | 7 (2.9%) | 10 (4.1%) | 0.6228 |
| Pressure ulcer | 4 (1.7%) | 0 (0%) | 0.1235 |
| Traumatic fractures | 2 (0.8%) | 0 (0%) | 0.4990 |
| Drug adverse effects | 3 (1.2%) | 1 (0.4%) | 0.6234 |
| Nosocomial infections | 33 (13.6%) | 6 (2.5%) | <0.0001 |
| Pneumonia | 13 (5.3%) | 0 (0%) | 0.0002 |
| Urinary tract infection | 16 (6.6%) | 4 (1.7%) | 0.2505 |
| *Clostridioides difficile* colitis | 8 (3.3%) | 1 (0.4%) | 0.0373 |
| Sepsis | 0 (0%) | 1 (0.4%) | >0.9999 |
| Death in hospital | 32 (13.2%) | 19 (7.8%) |  |
| Mortality rate  Deaths / 100 patient days | 0.24 | 0.97 |  |
| Discharge destination |  |  |  |
| Acute care hospital | 5 (2.4%) | 2 (0.9%) | <0.0001 |
| Rehabilitation | 70 (33.2%) | 7 (3.1%) |  |
| Home | 14 (6.6%) | 91 (40.6%) |  |
| Home with community agency support | 52 (24.6%) | 90 (40.2%) |  |
| Retirement home | 16 (7.6%) | 21 (9.4%) |  |
| Nursing home | 34 (16.1%) | 3 (1.3%) |  |
| Chronic care | 15 (7.1%) | 4 (1.8%) |  |
| Other | 5 (2.4%) | 6 (2.7%) |  |

**References**

1. Rubin DB. The design versus the analysis of observational studies for causal effects: parallels with the design of randomized trials. *Stat Med*. 2007;26:20–36.

2. Austin PC. An introduction to propensity score methods for reducing the effects of confounding in observational studies. *Multivariate Behav Res.* 2011;46:399–424.

3. Austin PC. Balance diagnostics for comparing the distribution of baseline covariates between treatment groups in propensity score matched samples. *Stat Med.* 2009;28:3083–107.

4. Ho D, Imai K, King G, et al. MatchIt: Nonparametric Preprocessing for Parametric Casual Inference. <https://cran.r-project.org/web/packages/MatchIt/index.html>. Accessed 11 November 2019.
